# Cox-2 Negatively Affects the Protective Role of Propofol against Hypoxia/Reoxygenation Induced Cardiomyocytes Apoptosis through Suppressing Akt Signaling

**DOI:** 10.1155/2019/7587451

**Published:** 2019-07-16

**Authors:** Kangmu Ma, Jiapei Qiu, Mi Zhou, Yang Yang, Xiaofeng Ye

**Affiliations:** Department of Cardiac Surgery, Ruijin Hospital, Shanghai Jiao Tong University School of Medicine, Shanghai 200025, China

## Abstract

Nowadays, the prevention of severe myocardium injury resulting from myocardial ischemia/reperfusion injury (I/R) has been recognized as an important subject in the field of ischemic heart disease. In this study, H9c2 cardiomyocytes were exposed to cycles of hypoxia/reoxygenation (H/R) to mimic myocardial I/R injury. Western blot analysis and qRT-PCR were performed to detect the expression of Cox-2, Akt and p-Akt. Cell viability, LDH release and activity of Caspase-3 were assessed to determine the protective effect of propofol. The results proved that the protective effect of propofol for H/R challenged cardiomyocytes was associated with Akt phosphorylation. We also revealed that treatment of propofol suppressed the expression of Cox-2 in cardiomyocytes which was up-regulated after H/R treatment. Conversely, the over-expression of Cox-2 inhibited Akt phosphorylation while enhancing cardiomyocytes apoptosis. Interestingly, Akt activator exhibited similar protective effect with propofol and could diminish the influences brought by over-expression of Cox-2. Thus, it could be concluded that Cox-2 negatively affects the protective effect of propofol against hypoxia/reoxygenation induced cardiomyocyte apoptosis by suppressing Akt phosphorylation.

## 1. Introduction

Nowadays, the ischemic heart disease, which is usually resulting from partial or complete blockage of coronary arteries, has been recognized as a leading cause of death in patients with cardiovascular diseases worldwide, especially in developed countries [1]. It is well known that revascularization is the most effective method to treat ischemic heart disease by restoring the coronary blood flow [2]. The improvement of the patient survival rate by coronary reperfusion has been clinically proved. However, reperfusion itself also shows the potential to cause lethal myocardium injury, a process termed “myocardial ischemia/reperfusion (I/R) injury” [3]. Clinically, myocardial I/R injury could induce the expansion of the myocardial infarct area, cardiac arrhythmias, contractile dysfunction and even sudden death [4, 5]. Therefore, it is of profound clinical significance to understand the mechanism of myocardial I/R injury for exploring more effective therapies.

The effort at alleviating myocardial I/R injury has continued for over 50 years. Jennings* et al*. first described myocardial I/R injury using a canine heart coronary artery ligation model in 1960 [6]. It showed that the development of myocardial necrosis was distinctly accelerated after reperfusion. The degree of myocardial necrosis 30-60 min post I/R was similar to that observed 24 h after coronary occlusion. Although several factors, such as reactive oxygen species (ROS) formation [7–9], calcium overload [10] and inflammatory responses [11], have been proved to be involved, mechanisms mediating the myocardial I/R injury on molecular level are still not fully understood.

Protein kinase B (PKB), also known as Akt, is a serine/threonine-specific protein kinase that plays a key role in multiple cellular processes such as glucose metabolism, apoptosis, cell proliferation, transcription and cell migration [12, 13]. Accumulating evidence has revealed the protective role of Akt activation during myocardial I/R injury [14]. Activation of the anti-apoptotic signaling pathway PI3K/Akt could regulate Bcl-2 and inhibit Caspase cascade activation and death protein expression [15]. On the other hand, cyclooxygenase-2 (Cox-2), one isoform of cyclooxygenase which catalyzes the transformation of arachidonic acid to prostanoids [16], has been recognized as a protein highly expressed in the cardiac tissue during myocardial ischemia and proved to associate with the severity of apoptosis in myocardial infarction [17]. Although a previous work reported the myocardial protective effect of Cox-2 on myocardial I/R animal model [18], much more studies have shown that it plays a harmful role in the process of myocardial I/R, and its inhibition could play a protective role for cardiomyocytes [19].

As previously reported, propofol (2,6-diisopropylphenol), a widely used intravenous anesthetic, was found to deliver protection in animal models against myocardial I/R injury caused by free radical consuming [20–22]. However, the underlying mechanism of the cardioprotective effect of propofol is still largely unknown. In this study, cycles of hypoxia/reoxygenation (H/R) exposure were employed to mimic I/R challenge and H9c2 cardiomyocytes were used as a cell model to investigate the effect and mechanism of propofol on H/R induced cardiomyocyte apoptosis. Akt phosphorylation and Cox-2 expression were investigated to illustrate their roles in propofol-induced myocardial protection under H/R challenge.

## 2. Materials and Methods

### 2.1. Cell Culture

H9c2 embryonic rat heart-derived cells from ATCC, at passages 5 to 10, were maintained at 37°C and 5% CO_2_ in Dulbecco's modified Eagle's medium supplemented with 10% (v/v) fetal bovine serum (Gibco, Grand Island, NY, USA), penicillin (100 units/mL) and streptomycin (100 mg/mL, Invitrogen, Carlsbad, CA, USA) on cells plated in 6-well tissue culture plates with a density of 2 × 10^6^ cells per well following the methods of Deng* et al.* 2017 [21]. The SC79 (2-Amino-6-chloro-*α*-cyano-3-(ethoxycarbonyl)-4H-1-benzopyran-4-acetic acid ethyl ester, Akt activator) used in the treatment of H9c2 cells were purchased from Selleck (Beijing, China).

### 2.2. Hypoxia/Reoxygenation (H/R) Challenge

We followed the methods of Deng* et al.* 2017 to perform H/R challenge [21]. A closed plastic vessel was used to create a moist and hypoxic environment. Cell cultures were primed in the hypoxic vessel, filled with a mixture of 94% N_2_, 5% CO_2_ and 1% O_2_ for 5 minutes under 37°C, and subjected to such hypoxic condition for 12 h then reoxygenated for 6 h by moving cells to the cell incubator with a normal culture condition (37°C, 5% CO2 and 5% O_2_). The cells were assigned to the following groups: Negative control (NC) group which was cultured in normal medium + 10% fetal bovine serum (FBS) and did not undergo H/R; positive control group which underwent H/R, and propofol treated groups in which cells were treated with various concentrations of propofol (i.e., P12.5 (12.5 *μ*M), P25 (25 *μ*M), P50 (50 *μ*M), P100 (100 *μ*M)) or the solvent DMSO group (DMSO at 100 *μ*M) respectively during reoxygenation for 6 h.

### 2.3. Cell Viability Measured by CCK-8 Assay

Cell viability was determined using a CCK-8 assay kit (Dojindo Laboratories, Kumamoto, Japan) according to the manufacturer's instructions. The principle of this assay is that some components of the CCK-8 assay kit will be reduced by mitochondria to produce formazan so as to be detectable. OD values resulting from CCK-8 staining and from mitochondrial viability staining were measured using a microplate reader at the wavelength of 450 nm.

### 2.4. Lactate Dehydrogenase Detection

Lactate dehydrogenase (LDH) is a glycolytic enzyme involved in pyruvate to lactic acid metabolism, which presents in almost all tissues or cytoplasm in the body. When the cell membrane damages, LDH releases rapidly. The detection of LDH was performed following the methods of Deng* et al.* 2017 [21]. The degree of cell damage was determined by detecting LDH activity in cell culture supernatant using a commercial LDH kit (Roche, Mannheim, Germany). Cell culture medium was processed and the OD value was measured using a microplate reader at the wavelength of 450 nm.

### 2.5. Plasmids and Transfection

Rat Cox-2 cDNA was amplified from H9c2 cells by reverse transcription-polymerase chain reaction (RT-PCR; Forward primer: 5'-GGGGTACCGCCACCATGCTCTTCCGAGCTGTG-3' and reverse primer: 5' -CCGCTCGAGTTACAGCTCAGTTGAACGCC-3'). The PCR products were digested with Kpn I and Xhol I (New England Biolabs, Beijing, China) and ligated with T4 ligase (New England Biolabs, Beijing, China) into pcDNA3.1(+) vectors to generate the Cox-2 constructs for transfection. 2 × 10^5^ H9c2 cells per well in six well tissue culture plate were cultured in an incubator with DMEM+10% FBS under the condition of 37°C, 5% CO_2_ and 95% O_2_ overnight and were then transfected with plasmids using Lipofectamine®2000 (Invitrogen; Thermo Fisher Scientific, MA, USA) according to the manufacturer's instructions.

### 2.6. Real-Time PCR

Total RNA from H9c2 cells was extracted using the Trizol reagent (Invitrogen, Carlsbad, USA). Complementary DNA (cDNA) was synthesized using Takara RNA PCR kit (Takara Bio, Inc., Otsu, Japan) according to the manufacturer's instructions. Real-time PCR was performed for quantification of Cox-2 expression using a quantitative thermal cycler (Millipore, Billerica, MA, USA). Sets of PCR primers for COX-2 are as follows: sense 5′-ATTGCTGGCCGGGTTGCTGG-3′ and antisense 5′-TCAGGGAGAAGCGTTTGCGGT-3′; *β*-actin: sense 5′-TCACCCACACTGTGCCCCATCTACGA-3′ and antisense 5′ CAGCGGAACCGCTCA TTGCCAATG G-3′. Relative gene expression was determined by delta-delta CT method (ABI, Applied Biosystems, MA, USA) with *β*-actin as the endogenous control, and the cycling program was conducted as follows: 50°C for 2 min, 95°C for 10 min and subsequent forty cycles of 95°C for 15 s, 60°C for 1 min. All data were analyzed using GraphPad Prism 5 software.

### 2.7. Western Blot

After the indicated treatments, H9c2 cells were harvested and lysed by using RIPA lysis buffer (Cell Signal Technology, MA, USA), and the homogenate was centrifuged at 12,000 rpm for 10 min at 4°C in a centrifuge (Beckman Coulter, CA, USA). A Takara BCA Protein Assay Kit (cat. no. T9300A; Takara Bio, Inc., Otsu, Japan) was used to detect the total protein concentrations. Total protein (30 *μ*g from each sample) was separated by 12% sodium dodecyl sulfate-polyacrylamide gel electrophoresis (SDS-PAGE). The protein in the gel was transferred to a polyvinylidene difluoride (PVDF) membrane (EMD Millipore, Billerica, MA, USA). The membrane was blocked with 5% free-fat milk in in TBS with Tween 20 (TBST) for 1 h at room temperature and incubated with Caspase-3, Cox-2, p-Akt (Thr-450), Akt, *β*-actin antibodies (Cell Signaling Technology, Danvers, MA, USA) in a dilution of 1:1,000. Following washing 3 times with TBST, these membranes were incubated with secondary antibodies, including IgG(H+L) horseradish peroxidase (HRP)-labeled goat anti-mouse IgG (cat. no. A0216) and IgG(H+L) HRP-labeled goat anti-rabbit IgG (cat. no. A0208), purchased from Beyotime Institute of Biotechnology (Jiangsu, China), for 2 h at room temperature at a dilution of 1: 10,000. *β*-actin served as the loading control. Finally, membranes were visualized using the enhanced chemiluminescence system (ECL; PerkinElmer Inc., Waltham, MA, USA).

### 2.8. Statistical Analysis

The data are expressed as mean ± SD (n ≥ 3) and analyzed using GraphPad Prism 6 software (GraphPad Software Inc., San Diego, CA, USA). The data were compared between two groups using two-tailed Student's t-test. P values less than 0.05 were considered statistically significant.

## 3. Results

### 3.1. Propofol Protected Cardiomyocytes from H/R Induced Apoptosis

**Figure 1:**
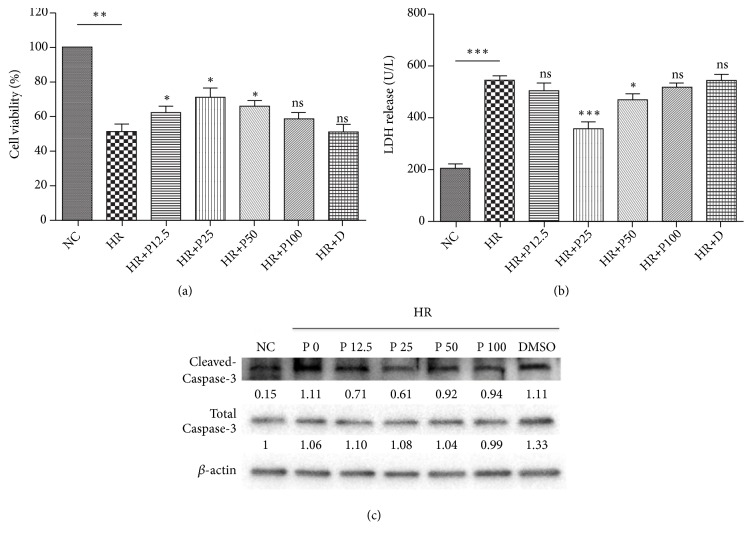
*Propofol protected cardiomyocytes from H/R induced apoptosis.* (a) Cell viability of H9c2 cardiomyocytes under H/R challenge with addition of propofol at different concentrations. (b) LDH release of H9c2 cardiomyocytes under H/R challenge with addition of propofol at different concentrations. (c) Western blot analysis of apoptosis related proteins (cleaved Caspase-3, total Caspase-3) in cardiomyocytes under H/R challenge with addition of propofol at different concentrations. NC means negative control; HR means H/R treatment group; P means treatment of propofol (in *μ*M); D means DMSO. Data were presented as the mean ± SD (n ≥ 3). *∗*P<0.05, *∗∗*P<0.01, *∗∗∗*P<0.001.

In order to investigate the protective effect of propofol against H/R injury, a protocol was established by culturing H9c2 cells under normoxic condition for 6 h after 12 h of hypoxia. The release of LDH, a biomarker for cell injuries, was measured and used to assess the cardiomyocyte viability in combination with CCK-8 assay. As shown in Figures 1(a) and 1(b), after the H/R cycle, drastic decrease in cell viability and increase in LDH release were detected by CCK-8 assay and LDH kit, respectively. On the other hand, treatment of propofol in various concentrations provided apparent protective effect, which is represented by the increase of cell viability and down-regulation of LDH release. Notably, propofol with the concentration of 25 *μ*M showed significant preservation of H9c2 cells from H/R-induced cell death, along with the most obvious decrease in LDH release (both P < 0.05). Furthermore, the suppressed expression of cleaved Caspase-3, a biomarker for apoptosis, measured by Western Blot analysis (Figure 1(c)), verified the H/R induced cardiomyocyte apoptosis and that the best efficacy of protective effect of propofol was at 25 *μ*M.

### 3.2. Propofol Inhibited the H/R Induced Up-Regulation of Cox-2 Expression under H/R Challenge

**Figure 2:**
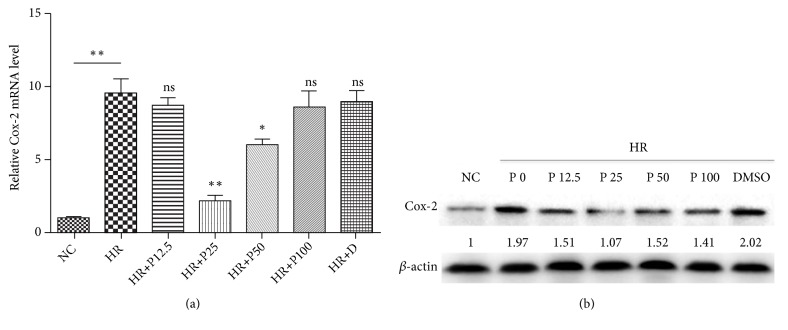
*Propofol inhibited the up-regulation of Cox-2 induced by H/R.* (a) Relative mRNA expression of Cox-2 in H9c2 cardiomyocytes under H/R challenge with addition of propofol at different concentrations. (b) Western blot analysis of Cox-2 expression in H9c2 cardiomyocytes under H/R challenge with addition of propofol at different concentrations. NC means negative control; HR means H/R treatment group; P means treatment of propofol (in *μ*M); D means DMSO. Data were presented as the mean ± SD (n ≥ 3). *∗*P<0.05, *∗∗*P<0.01, *∗∗∗*P<0.001.

Subsequently, the expression changes of Cox-2 induced by H/R were investigated by both qRT-PCR and Western Blot analysis. As demonstrated by Figure 2, expression of Cox-2 in both mRNA and protein levels was significantly up-regulated in cardiomyocytes after H/R treatment. Moreover, H/R-induced up-regulation of Cox-2 expression was also proved to be distinctly suppressed by propofol treatment, especially when at 25 and 50 *μ*M of propofol treatment.

### 3.3. Over-Expression of Cox-2 Inhibited the Protective Effect of Propofol

**Figure 3:**
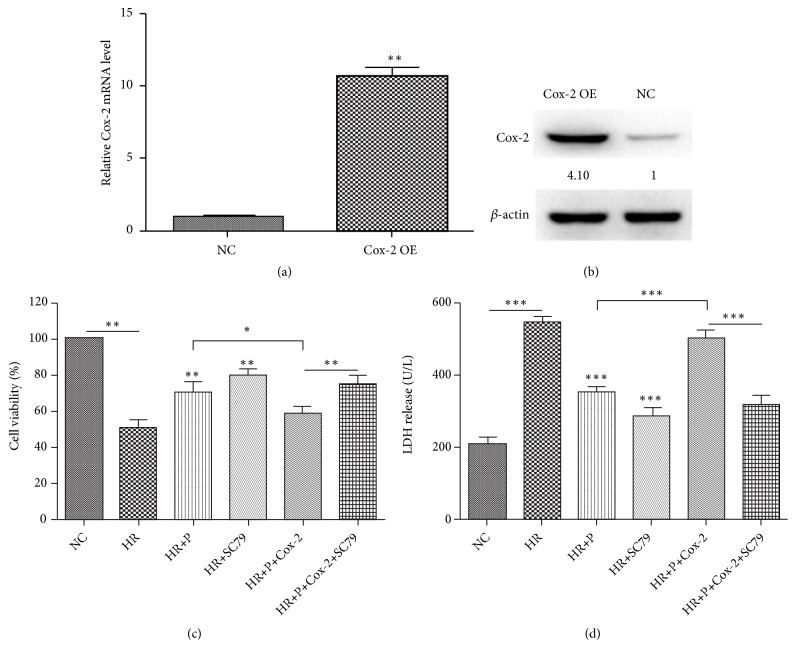
*Over-expression of Cox-2 suppressed the protective effect of propofol.* (a) Relative mRNA expression of Cox-2 in Cox-2 over-expressed H9c2 cardiomyocytes. (b) Western blot analysis of Cox-2 expression in Cox-2 over-expressed H9c2 cardiomyocytes. (c) Cell viability and (d) LDH release in H9c2 cardiomyocytes under indicated conditions. NC means negative control; OE means over-expression; HR means H/R treatment group; P means treatment of propofol; Cox-2 means Cox-2 over-expression; SC79 means treatment of SC79 (10 *μ*M). Data were presented as the mean ± SD (n ≥ 3). *∗*P<0.05, *∗∗*P<0.01, *∗∗∗*P<0.001.

**Figure 4:**
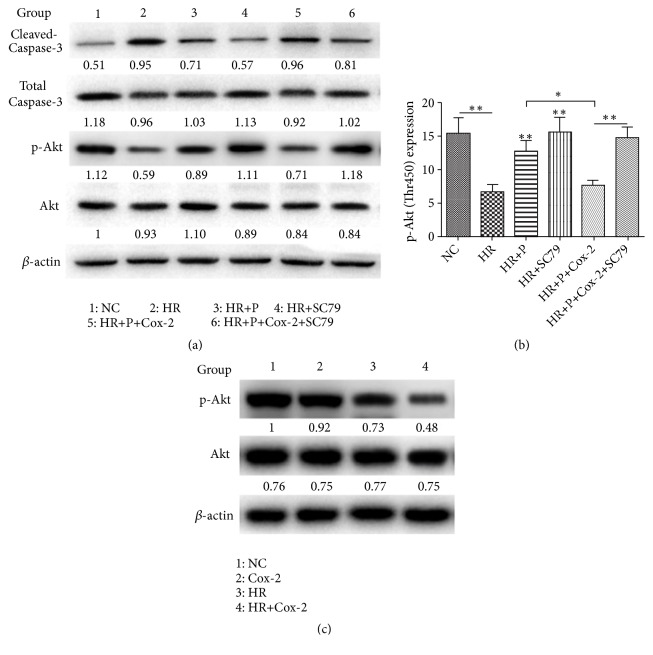
*Cox-2 suppressed Akt phosphorylation negatively affects the protective effect of propofol against hypoxia/reoxygenation induced cardiomyocyte apoptosis.* (a) Western blot analysis of cleaved Caspase-3, total Caspase-3, Akt and p-Akt under indicated conditions. (b) Expression of p-Akt (Thr450) under indicated conditions. (c) Western blot analysis of Akt and p-Akt under indicated conditions. NC means negative control; OE means over-expression; HR means H/R treatment group; P means treatment of propofol; Cox-2 means Cox-2 over-expression; SC79 means treatment of SC79 (10 *μ*M). Data were presented as the mean ± SD (n *⩾* 3). *∗*P<0.05, *∗∗*P<0.01.

The aforementioned results proved that propofol treatment could down-regulate H/R-induced Cox-2 expression in cardiomyocytes, along with an increase of cell viability. Subsequently, H9c2 cell model with Cox-2 over-expressed was further used to explore the role of Cox-2 in the myocardial protective effect of propofol. Cox-2 over-expression was confirmed by qRT-PCR and Western blot analysis (Figures 3(a) and 3(b)). As shown in the CCK-8 assay (Figure 3(c)) and LDH measurement (Figure 3(d)), the protective effect of propofol against H/R injury was inhibited to a large extent in cardiomyocytes with Cox-2 over-expression, which was also proved through the cleaved/total Caspase-3 measurement by Western blot analysis (Figure 4(a)).

### 3.4. Up-Regulation of Akt Phosphorylation by Propofol Could Be Inhibited by Cox-2

**Figure 5:**
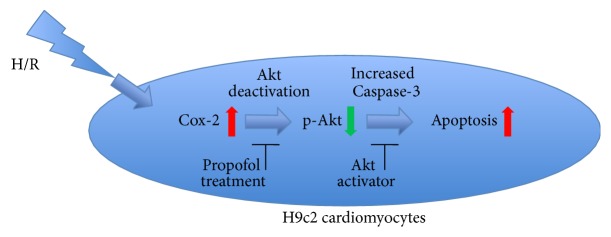
*Schematic program of mechanism.* The proposed mechanism of regulation ability of Cox-2/Akt cascade in the H/R induced cardiomyocytes apoptosis and the protection effect of propofol.

Accumulating evidence has shown that the Akt signaling pathway plays an important role in controlling cardiomyocytes survival and apoptosis [23]. In order to investigate whether the mechanism of the protective effect of propofol to cardiomyocytes against H/R injury involves the activation of Akt, the protein expression levels of total Akt and p-Akt (Thr450) were examined* via* Western blot analysis. Comparison between the negative control group and H/R group revealed that, with approximately same expression of total Akt, expression of p-Akt decreased in the H/R group (Figures 4(a) and 4(b)). More importantly, the suppression of Akt phosphorylation could be distinctly relieved by propofol treatment while such effect was almost completely eliminated by Cox-2 over-expression (Figures 4(a) and 4(b)), which was also in accordance with the inhibition effects of Cox-2 over-expression on p-Akt under normal and H/R conditions (Figure 4(c)) [19]. In order to further investigate the relationship between Cox-2 and Akt phosphorylation, the Cox-2 over-expressed H9c2 cells were treated with Akt activator (SC79, 10 *μ*M). As shown in Figures 3 and 4, except for the up-regulation of p-Akt, the influences of the cell viability and LDH release as well as expression of cleaved Caspase-3 by Cox-2 over-expression were almost diminished by the activation of Akt. Moreover, for the H9c2 cells treated with only H/R cycles, the treatment of SC79 exhibited similar protective effect with propofol, represented by increase of cell viability and decrease in LDH release as well as expression of cleaved Caspase-3, and the reversion of the H/R induced up-regulation of Cox-2 expression. These results revealed the regulation ability of Cox-2/Akt cascade in the H/R induced cardiomyocytes apoptosis and the protection effect of propofol, which is generally presented as Figure 5.

## 4. Discussion

Ischemic heart disease causes myocardial ischemia and hypoxia injury due to reduced oxygen supply from coronary blood flow. It is a fatal factor that threatens human health and life. Although revascularization and coronary reperfusion therapy has significantly reduced the mortality of ischemic heart disease [24], it also brings unwanted extra tissue damage, termed as myocardial I/R injury [4]. After the first proposal of myocardial I/R injury by Jennings* et al.* [6], it has been classified into 4 types according to the degree of cardiac dysfunction: myocardial stunning, no reflow of myocardium, reperfusion arrhythmias and lethal reperfusion injury. It has been reported that about 50% of myocardial infarction area is caused by myocardial I/R injury, and the rational intervention of myocardial I/R injury can maximize the effect of reperfusion therapy [25]. Moreover, accumulating evidence has indicated that apoptosis of cardiomyocyte plays particularly important role in myocardial I/R injury, which could aggravate I/R injury and affect the size of infarction as a major factor [26, 27]. Wu* et al.* reported that the inhibition of apoptosis was a key factor in the Osthole-induced attenuation of myocardial I/R injury in rat models [28]. Zhou* et al.* also indicated that Apigenin could execute its protective role in myocardial I/R injury through suppressing apoptosis of H9c2 cells [29]. Indeed, our study, in which H/R challenged H9c2 cell model was used to mimic myocardial I/R injury, showed high level of apoptosis, as well as low level of cell viability, in H9c2 cells post H/R treatment, indicating the harmful effect during reperfusion. The treatment strategy for alleviating myocardial I/R injury is still in urgent need, and this research has important clinical significance for patients with ischemic heart disease.

Propofol is a commonly used clinical anesthetic. Previous studies have shown that it can combine with oxygen free radicals to produce stable phenoxy free radicals, act as an antioxidant and thus protecting the I/R injury of organs such as liver and kidney [30, 31]. Li* et al.* reported that propofol could inhibit H_2_O_2_-induced injury in H9c2 cells via decreasing NF-*κ*B activation and PUMA expression, thus improving cell survival. Moreover, the protective effect of propofol in H/R injury of cardiomyocytes has also been proved [21, 22, 32]. For example, Deng et al. reported that treatment of propofol could up-regulate Caveolin-3 expression, thus alleviating mitochondrial damage and H/R injury of H9c2 cardiomyocytes [21]. However, the underlying mechanism of the myocardial protection effect of propofol is still largely unknown and rarely reported. On the other hand, it was also proved that the protective effect provided by propofol for cardiomyocyte is dose-dependent [21, 32]. Therefore, in our study, several clinically achievable propofol concentrations from 12.5 *μ*M to 100 *μ*M were chosen for experiment. Our research exhibited that propofol at 25 *μ*M generated maximal protective effect on H9c2 cells against H/R injury, while some studies showed that propofol at concentrations of 50 *μ*M or 100 *μ*M provided better protective effect on cells in case of I/R [33, 34]. We speculated that the differences might be ascribed to several factors, such as cell model, cell type and experimental environment.

Akt is a serine threonine kinase which plays important role in cell proliferation and survival [35]. Acute activation of Akt could inhibit necrosis and apoptosis of cardiomyocytes induced by deleterious stimuli [36]. Jiang* et al.* indicated that salidroside exhibited cardioprotective effect for H9c2 cardiomyocytes during peroxide-induced injury via PI3K/Akt dependent pathway [37]. As mentioned before, it has been revealed that the activation of Akt during myocardial I/R injury could provide cardioprotective effects for cardiomyocytes [14]. More directly, Qiu* et al.* demonstrated that shikonin could significantly enhance the phosphorylation of Akt and GSK-3*β* in H/R treated H9c2 cells, which could be reversed by a specific PI3K/Akt inhibitor [23]. Our results also demonstrated that the underlying mechanism of propofol-induced cardioprotective effects during H/R involved the phosphorylation of Akt. In this study, with similar expression of total Akt, the expression of p-Akt, which is essential in the protective signaling pathway in cardiomyocytes, was much higher in the propofol treated group. The results suggested that propofol may execute its cardioprotective effect through the phosphorylation of Akt. Actually, this conclusion could be further proved by the results that treatment of Akt activator SC79 exhibited similar protective effect to propofol.

Cox-2 is an inducible enzyme, which can be transcribed and translated rapidly under the stimulation of external conditions such as I/R process [38]. In addition to regulating inflammation, Cox-2 is also involved in the occurrence and metastasis of tumors and plays a variety of roles in cardiovascular and cerebrovascular diseases [39]. Despite of the controversy, accumulating evidence suggested that Cox-2 plays a harmful role during myocardial I/R injury [40]. Herein, our results also proved the significantly enhanced Cox-2 expression in H/R treated H9c2 cardiomyocytes which might suggest the up-regulation of Cox-2 expression could be a potential change mediating myocardial I/R injury. This possible mechanism was further proved by the inhibition of the cardioprotective effect of propofol by Cox-2 over-expression. Moreover, the regulation of cell viability, LDH release, expression of cleaved Caspase-3 and activation of Akt by Cox-2 over-expression were found to be reversed to a large extent by the treatment of Akt activator, suggesting a possible Cox-2/Akt regulation cascade during process. Interestingly, it is commonly reported that Cox-2 activation was positively associated with Akt phosphorylation and poor survival in cancer research [41, 42]. Nevertheless, in this study, contrary results were obtained in H9c2 cardiomyocytes, implying a totally different mechanism in cardiomyocytes from cancer cells. Despite of all the above results, this study was still limited by the lacking of experiments in animal models, which would be further improved in our future work.

## 5. Conclusions

In conclusion, this study showed that Cox-2 could negatively influence the protective effect of propofol against H/R-induced cardiomyocyte apoptosis by suppressing Akt phosphorylation. This study can provide theoretical basis and new targets for the treatment of myocardial I/R injury and is of significant importance for the development of myocardial I/R injury therapy.

## Data Availability

The data used to support the findings of this study are available from the corresponding author upon request.
